# Editorial: Brain injury associated secondary injury and remote organ injury

**DOI:** 10.3389/fnins.2024.1398800

**Published:** 2024-04-02

**Authors:** Lujia Tang, Kaibin Shi, Yanjun Zhang, Ying Fu, Jie Gao, Zilong Zhao

**Affiliations:** ^1^Department of Neurosurgery, Tianjin Institute of Neurology, Tianjin Medical University General Hospital, Tianjin, China; ^2^Department of Neurology, China National Clinical Research Center for Neurological Diseases, Beijing Tiantan Hospital, Capital Medical University, Beijing, China; ^3^Chinese Institutes for Medical Research, Beijing, China; ^4^Nano Life Science Institute, Kanazawa University, Kanazawa, Japan; ^5^Fujian Key Laboratory of Molecular Neurology, Department of Neurology, Institute of Neurology of First Affiliated Hospital, Institute of Neuroscience, Fujian Medical University, Fuzhou, China; ^6^State Key Laboratory of Medicinal Chemical Biology, Key Laboratory of Bioactive Materials, Ministry of Education, College of Life Sciences, Nankai University, Tianjin, China; ^7^State Key Laboratory of Experimental Hematology, Tianjin, China

**Keywords:** brain injury, secondary injury cascade, neurological disorder, diagnosis and targeted therapy, emerging technology

Brain injury including neurotrauma, neurodegenerative disease, stroke and other brain disorders remains a threat to human health. The cascade events caused by the primary injury lead to remote organ secondary injury and damage deterioration, and the underlying cellular and molecular mechanisms remain to be elucidated. Numerous studies reveal potential mechanisms including a deregulated inflammatory response, autoimmune disorders, and pathological molecules such as damage-associated molecular patterns (DAMPs). However this field still needs further investigation to exploit innovative therapeutic strategies for various complicated pathological conditions. With emerging technologies such as bioinformatics, neuroimaging, and RNA sequencing, interdisciplinary joint research has become dominant. Therefore, neuroscientists need to implement multi-omics analysis through multi-partner collaboration and close communication to obtain more novel therapeutic strategies. In this collection of works, we present six studies focusing on different aspects of brain injury with emerging techniques.

The first article by Huang et al. identified the exosome existing in the serum of traumatic brain injury patients and revealed the correlation of exosomal miRNA with pathological progression after intracerebral hemorrhage. The authors analyzed the differential expression of exosomal miRNA. Using next-generation sequencing, they found 245 significantly altered miRNAs that were consistent with specific biological processes based on their known functions. This study suggested that miRNA target gene levels are connected with various secondary injury cascades, indicating the broad insight of exosomal miRNA as an accurate indicator and therapeutic application target.

Massive ischemic stroke (MCI), with its high morbidity and mortality, is a cerebrovascular disease in need of treatment. Guo et al. identified Myd88 and Ccl3 as key genes for the propagation of inflammation after ischemic injury. TWS-199′s unlimited potential for future therapeutic intervention through the use of bioinformatics. The researchers identified 215 differentially expressed genes through microarray data from a mouse model of ischemic stroke and selected the most abundant pro-inflammatory pathways. In particular, Myd88 and Ccl3 were identified as hub genes and central to the pathophysiology of ischemic injury. CMap analysis indicates that the compound TWS-199 may be a potential therapeutic agent for the promising treatment of MCI. This study deepens our understanding of the molecular mechanisms of MCI and offers new avenues for ameliorating the disease.

The progress and prospect of clinical therapy, acupuncture applied to post-stroke cognitive impairment (PSCI), were described by Li et al.. They revealed the benefits and concrete mechanisms of acupuncture in the treatment of PSCI. The study showed the exact influencing aspects of acupuncture, such as neuroprotection, anti-inflammation, improvement of synaptic plasticity, and regulation of brain energy metabolism. Strong evidence from many studies consolidates the beneficial status of acupuncture, highlighting its effect on pathway regulation, neurotransmitter systems and molecular pathways. This study proposed acupuncture as an effective treatment modality for improving PSCI, and provided scientific proof for it.

The impact of the β-receptor blocker propranolol and its treatment effect on bone marrow following mild traumatic brain injury (mTBI) in mice was reported by Smith et al.. The study demonstrated a remarkable change in the transcriptome of bone marrow tissue, related to propranolol treatment and distinct time points post-injury. This work revealed that propranolol may impact the bone marrow response by regulating a series of pathways, including proinflammation, metabolism and the cell cycle. This study presented an insight into the use of propranolol in the management of the systematic damage brought by TBI in terms of bone marrow function and response to injury, and helped to better understand the mechanism of injury events following TBI.

To understand the critical role of mitochondria in neurological disorders, Lu et al. provided a comprehensive interpretation of the mitochondrial transport pathway. The authors emphasized its pivotal function in mitochondrial quality control and its complications in various acute neurological disorders. As unavoidable components involved in mitochondrial transport, the detailed types and influence of motors and adaptors were elucidated accurately. The authors highlighted mechanisms of mitochondrial transport regulation and the impact of mitochondrial local disposition. They also explored mitochondrial dynamics and alternation under mechanical stress, suggesting that disorder in mitochondrial transport contributes to the aggravation of diseases.

The pathophysiology of sepsis-associated encephalopathy (SAE) still remains unclear. Kakizaki et al. confirmed the effect of sepsis on hippocampal long-term potentiation (LTP) in rats. By establishing a sepsis-induced rat model, they observed enhanced somatic excitatory and altered excitatory synaptic transmission in late sepsis by measuring LTP in hippocampal slices at different time points post-injury. A free radical scavenger, superoxide dismutase (SOD) was determined to be a treatment that could inhibit pre-CLP disruption. The findings shed further light on the neural mechanisms underlying sepsis-associated encephalopathy and revealed the potential role of oxidative stress in these processes.

Together, these six papers provide a comprehensive overview of brain injury and identify a number of novel mechanisms at work in the progression of various neurological diseases. The findings from these papers accelerate our understanding and unveil potential diagnostic markers and therapeutic strategies.

## Author contributions

LT: Writing – review & editing. KS: Writing – original draft. YZ: Writing – original draft. YF: Writing – original draft. JG: Writing – original draft. ZZ: Writing – review & editing.

